# Pharmacokinetics of Pegaspargase with a Limited Sampling Strategy for Asparaginase Activity Monitoring in Children with Acute Lymphoblastic Leukemia

**DOI:** 10.3390/pharmaceutics17070915

**Published:** 2025-07-15

**Authors:** Cristina Matteo, Antonella Colombini, Marta Cancelliere, Tommaso Ceruti, Ilaria Fuso Nerini, Luca Porcu, Massimo Zucchetti, Daniela Silvestri, Maria Grazia Valsecchi, Rosanna Parasole, Luciana Vinti, Nicoletta Bertorello, Daniela Onofrillo, Massimo Provenzi, Elena Chiocca, Luca Lo Nigro, Laura Rachele Bettini, Giacomo Gotti, Silvia Bungaro, Martin Schrappe, Paolo Ubezio, Carmelo Rizzari

**Affiliations:** 1 Laboratory of Cancer Pharmacology, Department of Oncology, Istituto di Ricerche Farmacologiche Mario Negri IRCCS, 20156 Milan, Italy; cristina.matteo@marionegri.it (C.M.); marta.cancelliere@marionegri.it (M.C.); tommasogiacomo.ceruti@bpt.eurofinseu.com (T.C.); ilaria.fusonerini@humanitasresearch.it (I.F.N.); luca.porcu@cruk.cam.ac.uk (L.P.); paolo.ubezio@marionegri.it (P.U.); 2 Department of Pediatrics, Fondazione IRCCS San Gerardo dei Tintori, 20900 Monza, Italy; antonella.colombini@irccs-sangerardo.it (A.C.); laurarachele.bettini@irccs-sangerardo.it (L.R.B.); giacomo.gotti@irccs-sangerardo.it (G.G.); silvia.bungaro@irccs-sangerardo.it (S.B.); carmelo.rizzari@irccs-sangerardo.it (C.R.); 3 Tettamanti Center, Fondazione IRCCS San Gerardo dei Tintori, 20900 Monza, Italy; daniela.silvestri@unimib.it; 4 Biostatistics and Clinical Epidemiology, Fondazione IRCCS San Gerardo dei Tintori, 20900 Monza, Italy; grazia.valsecchi@unimib.it; 5 School of Medicine and Surgery, University of Milan-Bicocca, 20126 Monza, Italy; 6 Dipartimento di Oncologia, Ematologia e Terapie Cellulari, AORN Santobono-Pausilipon, 80122 Napoli, Italy; r.parasole@santobonopausilipon.it; 7 Dipartimento di Onco-Ematologia e Terapia Cellulare e Genica, Ospedale Pediatrico Bambino Gesù, 00050 Rome, Italy; luciana.vinti@opbg.net; 8 Oncoematologia Pediatrica, Ospedale Infantile Regina Margherita, Città della Salute e della Scienza, 10126 Torino, Italy; nicoletta.bertorello@unito.it; 9 Oncoematologia Pediatrica, Dipartimento di Oncologia e Ematologia, Ospedale Civile Spirito Santo, 65100 Pescara, Italy; daniela.onofrillo@asl.pe.it; 10 Oncologia Pediatrica, Ospedale Papa Giovanni XXIII, 24127 Bergamo, Italy; mprovenzi@asst-pg23.it; 11 Department of Hematology-Oncology, Meyer Children’s Hospital IRCCS, 50139 Florence, Italy; elena.chiocca@meyer.it; 12 Centro di Ematologia ed Oncologia Pediatrica, Azienda Ospedaliero Universitaria, Policlinico “G. Rodolico-San Marco”, 95100 Catania, Italy; lonigro@policlinico.unict.it; 13 Department of Medicine and Surgery, University of Milano-Bicocca, 20126 Milan, Italy; 14 Department of Paediatrics, Christian-Albrechts-University Kiel and University Medical Centre Schleswig-Holstein, 24118 Kiel, Germany; m.schrappe@pediatrics.uni-kiel.de

**Keywords:** asparaginase (ASPase), L-asparagine (Asn), pegylated asparaginase (PEG-ASPase), pharmacokinetics (PK), limited sampling strategy, acute lymphoblastic leukemia (ALL), serum ASPase activity (SAA)

## Abstract

**Background**: Asparaginase (ASPase) plays an important role in the therapy of acute lymphoblastic leukemia (ALL). Serum ASPase activity (SAA) can be modified and even abolished by host immune responses; therefore, current treatment guidelines recommend to monitor SAA during treatment administration. The SAA monitoring schedule needs to be carefully planned to reduce the number of samples without hampering the possibility of measuring pharmacokinetics (PK) parameters in individual patients. Complex modelling approaches, not easily applicable in common practice, have been applied in previous studies to estimate ASPase PK parameters. This study aimed to estimate PK parameters by using a simplified approach suitable for real-world settings with limited sampling. **Methods**: Our study was based on 434 patients treated in Italy within the AIEOP-BFM ALL 2009 trial. During the induction phase, patients received two doses of pegylated ASPase and were monitored with blood sampling at five time points, including time 0. PK parameters were estimated by using the individually available SAA measurements with simple modifications of the classical non-compartmental PK analysis. We also took the opportunity to develop and validate a series of limited sampling models to predict ASPase exposure. **Results**: During the induction phase, average ASPase activity at day 7 was 1380 IU/L after the first dose and 1948 IU/L after the second dose; therapeutic SAA levels (>100 IU/L) were maintained until day 33 in 90.1% of patients. The average AUC and clearance were 46,937 IU/L × day and 0.114 L/day/m^2^, respectively. The database was analyzed for possible associations of PK parameters with biological characteristics of the patients, finding only a limited dependence on sex, age and risk score; however, these differences were not sufficient to allow any dose or schedule adjustments. Thereafter the possibility of further sampling reduction by using simple linear models to estimate the AUC was also explored. The most simple model required only two samplings 7 days after each ASPase dose, with the AUC being proportional to the sum of the two measured activities A(7) and A(21), calculated by the formula AUC = 14.1 × [A(7) + A(21)]. This model predicts the AUC with 6% average error and 35% maximum error compared to the AUC estimated with all available measures. **Conclusions**: Our study demonstrates the feasibility of a direct estimation of PK parameters in a real-life situation with limited and variable blood sampling schedules and also offers a simplified method and formulae easily applicable in clinical practice while maintaining a reliable pharmacokinetic monitoring.

## 1. Introduction

The enzyme asparaginase (ASPase) is an essential drug in the treatment protocols for acute lymphoblastic leukemia (ALL), particularly in children and young adults [1,2,3]. ASPase hydrolyses the amino acid L-asparagine (Asn) in serum to ammonia and L-aspartic acid, which in turn results in a depletion of the circulating pool of Asn [4]. Asn is a non-essential amino acid required for cell survival. Normal healthy cells are capable of synthesizing their own Asn, but malignant lymphoblasts have a reduced or null expression of the asparagine synthetase enzyme and thereby depend on a necessary supply of Asn from extracellular sources [5,6]. Asn depletion leads to leukemic cell death so that the addition of ASPase to leukemia treatment contributes to the known recent increase of the cure rate up to 85–90% [7,8,9]. ASPase is usually well tolerated but also has some relevant side effects, including hypersensitivity reactions (HSRs), pancreatitis and venous thromboembolism [10]. HSRs are caused by the production of antibodies, often resulting in a complete neutralization of the drug. The clinically hidden version of the HSRs is called silent inactivation (SI), which consists of the neutralization of ASPase activity without clinical symptoms. Patients may also develop HSR symptoms without ASPase inactivation, i.e., allergic like reactions (ALRs) [11]. SI and ALRs can only be distinguished through the measurement of SAA levels. The monitoring of ASPase activity is therefore mainly performed to optimize ASPase therapeutic efficacy. As said, the aim of ASPase treatment is to achieve and to maintain a complete and long-lasting Asn depletion; it is commonly accepted that maintaining systemic serum ASPase activity levels above a given threshold represents a valid surrogate marker of Asn depletion. Based on data mainly obtained with the native *Escherichia coli* ASPase, this threshold has been set at 100 IU/L [12,13], an activity level which should be maintained throughout the whole planned treatment period. This threshold has been shown to cause Asn depletion in serum and limited depletion in the cerebrospinal fluid (CSF) [14,15,16]. However, some data also suggest that SAA trough levels below 100 IU/L may result in sufficient serum Asn depletion [17,18,19]. Moreover, different ASPase preparations derived from *E. coli* or from *Erwinia chrysanthemi* have been progressively introduced into the market from the seventies onwards [6,20]. Some of them show pharmacokinetic (PK) profiles very different from those associated with the native *E. coli* ASPase and are administered with different schedules, with possible different effects on the time-course of Asn depletion. Among the new preparations, the pegylated forms of native asparaginase represent nowadays the first-line treatment products of pediatric ALL in developed countries. Pegylation decreases the immunogenicity and increases the half-life of the bulk product. We studied the PK parameters associated with pegaspargase (PEG-ASPase, Oncaspar^®^ [21,22]) in a large number of children enrolled in the Italian AIEOP (Italian Association of Pediatric Hematology and Oncology) centers participating in the clinical trial AIEOP-BFM ALL 2009, wherein it was chosen as first line ASPase product. In that trial, tight SAA monitoring was implemented to evaluate the presence of adequate therapeutic levels of the enzyme and to rule out any drug inactivation which could lead to its substitution with the *E. chrysanthemi* ASPase product [12]. Current guidelines on asparaginase activity monitoring are reported in a very recent publication [23]. Beside ASPase activity levels at specific time points, the PK parameters could in principle give relevant information for schedule optimization, but their measure encounters challenges that seem unaffordable with the limited sampling protocols in use.

The AIEOP-BFM ALL 2009 protocol, in the induction phase, includes two PEG-ASP doses 14 days apart. SAA was monitored by planning five blood samples. With such a limited sampling schedule over a period exceeding one month it is difficult to estimate PK parameters on an individual basis; this puzzling aspect has been previously been approached by other investigators with population-based compartmental analyses [13,24,25,26,27,28]. Actually, the measure of PK parameters for individual patients raises a series of issues here examined, while also considering the real-world adherence with the planned five-samplings schedule. Our study aimed to estimate pharmacokinetic parameters, identifying simple mathematical corrections to the formulae of non-compartmental analysis and looking for relationships between the available ASPase activity measurements and the AUC, terminal elimination rate, days of permanence over 100 IU/L (T_>100_) and other parameters. This allowed us to analyze PEG-ASPase SAA measurements in 434 patients recruited in 27 Italian pediatric hematology-oncology units belonging to the AIEOP consortium.

## 2. Materials and Methods

### 2.1. Patients and Methods

From 1 October 2010 to 28 February 2017 a total of 2098 Italian patients, aged 1–17 years, were diagnosed with a Philadelphia chromosome negative ALL, and were enrolled in the AIEOP-BFM ALL 2009 Study (EudraCT number 2007-004270-43).

This protocol included PEG-ASPase given as a 2-h intravenous infusion, at the dose of 2500 IU/m^2^ capped at 3750 IU on days 12 and 26 of the induction phase.

A pharmacokinetic study was planned for all the patients recruited in the protocol. Blood samples were collected before (1 sample) and 7 and 14 days after each administration, plus one sample planned at a later time, as specified below.

Of the 2098 patients recruited in the protocol by the AIEOP centers, 560 had the planned blood sampling in induction. A total of 126/560 patients were not included in the cohort here described because of the following exclusion criteria:

- Additional PEG-ASPase administration in the subsequent phase IB (as per randomization planned in the protocol for HR patients (47 pts);
- The occurrence of an HSR episode (5 pts);
- The occurrence of an SI episode (9 pts);
- Insufficient numbers of measurement (52 pts), i.e., three or fewer measurements above the lower limit of quantification (LLOQ), or only one measurement >LLOQ after the second dose or no measurement between the two doses;
- Implausible measures (13 pts), i.e., with higher ASPase SAA at later time points compared to earlier time points, within the same administration.

Thus, 434 patients could be included in the pharmacokinetic study (Supplementary Figure S1).

According to ethical national guidelines, signed informed consent was obtained for each patient from their parents/guardians. The study protocol was approved by the national ethical committees, in accordance with the Declaration of Helsinki and national laws.

The distribution of the AIEOP patients’ characteristics is reported in Table 1.

This distribution was superimposable to the Czech and German cohort of patients who entered the study during the whole period of enrolment in the AIEOP-BFM ALL 2009 protocol, as elsewhere reported [24,29].

### 2.2. Pharmacokinetic Study Design

The scheduled times of the sample collections during the induction phase of the protocol were as follows: days 0 (before the first PEG-ASPase dose), 7, 14 (before the second PEG-ASPase dose), 21 and 28, (i.e., 7 and 14 days after the second PEG-ASPase dose), and at a subsequent time after day 30 (“reference schedule” in the following).

The actual collection times were recorded in dedicated CRF-PK forms and used for the pharmacokinetic calculations.

The second PEG-ASPase administration was given 14 days after the first one in 340/434 patients, 13 and 15–17 days in other 71 patients and it was delayed more than 3 days in the remaining 23 (5.3%) patients. The time of the last measurement was quite variable: in 248/434 (57.1%) patients it was done in the range of 31–35 days after the first PEG-ASPase dose, with day 33 being the most represented (74/434 pts), while in 43 patients (9.9%) the last sampling was performed on day 45 or later.

Blood samples were collected from a central venous catheter, in chilled tubes without anticoagulant and placed in a water/ice (4 °C) bath. After 60 min, the tubes were centrifuged at 2500 rpm at 4 °C for 5 min. After centrifugation, the serum was separated, collected and three aliquots were divided into as many polypropylene test tubes (sets A, B and C) and stored at −20 °C until analysis for the determination of the SAA.

### 2.3. Determination of SAA

SAA in the serum was assessed in a centralized certified Laboratory of Cancer Pharmacology at the Istituto di Ricerche Farmacologiche Mario Negri IRCCS, by using the enzymatic test MAAT (Medac Asparaginase Activity Test-Medac GmbH, Hamburg, Germany), which is an IVD-CE certified test [30]. It is a homogeneous microplate assay that analyses the PEG-ASPase catalytic activity in serum by detecting the amount of a hydrolyzed substrate (an analogue of Asn), quantified by photometric reading at 690 nm. The assay uses calibrators containing a native enzyme preparation from *E. coli* (ASPase, Medac) reaching a LLOQ of 30 IU/L. For pharmacokinetic elaboration and statistical analysis, the values below the LLOQ were considered LLOQ/2, i.e., 15 IU/L [31].

### 2.4. Pharmacokinetic Analysis

Previous reports of the PK of PEG-ASPase [17,32,33] showed that the time course of serum ASPase activity [A(t)] reaches a peak within 2 h of the IV administration, declines slowly later for up to 10 days, then enters a phase of terminal elimination with a more rapid decrease.

Since in the AIEOP-BFM ALL 2009 protocol a limited sampling schedule was planned for routine SAA monitoring, the methods of the calculation of the PK parameters as the terminal elimination rate constant (*k_el_*) and the area under the curve (AUC) were reassessed, mainly to reduce as much as possible the errors potentially introduced by an acritical application of the standard formulae.

#### 2.4.1. Determination of Terminal Elimination Rate and Half-Life

The terminal elimination rate could be estimated only after the second dose, with three measurements available. We initially considered alternative options for the estimate of *k_el_* by: (1) the best exponential fit of the three A(t) points and (2) the exponential interpolation of the last two points (at days “d_x_” and “d_y_”) with the formula:

*k_el_* = − ln (A(d_y_)/A(d_x_))/(d_y_ − d_x_) (1)

Option 2 was motivated by the consideration that only the two last measures were expected to fall within the terminal phase range according to previous reports [17,32,33].

Option 1 resulted in a lower estimate of *k_el_* in 89.4% instances (Supplementary Figure S2a), with an average 0.166 day^−1^ against the 0.214 day^−1^ with option 2. In fact, the decreasing trend of the activity calculated with the first two of the three available points was considerably lower (Supplementary Figure S2b), indicating that the beginning of the terminal phase occurs beyond 7 days after the administration. Thus, option 1 gives a systematic error of *k_el_*, which would impact also the extrapolation of the AUC to the infinite (see below). On the other hand, option 2 is expected to be more dependent on the precision of the two, instead of three, measures. In the attempt of avoiding a systematic underestimation error of option 1, we adopted option 2.

The second issue in the estimate of *k_el_* concerns the 69 cases where the last measure was under the LLOQ. In these cases, we made the calculation of *k_el_* as if that measure were equal to 15 IU/mL (=LLOQ/2). In eight instances, however, the last measure <LLOQ was considered non informative because the previous measure was already lower or equal to LLOQ. In these instances, *k_el_* was not calculated.

The terminal half-life (HL) was directly derived from *k_el_* as HL = ln(2)/*k_el_*.

#### 2.4.2. Determination of the AUC

The AUC is commonly estimated with the trapezoidal approximation until the last measure (AUC_last_) and extrapolated from that time to infinite assuming an exponential decrease with constant *k_el_* (AUC_inf_). In cases where the last measure was <LLOQ, the area after that time was neglected. Due to the variability of the time of the last measure, the AUC_last_ was not comparable among the patients, and we were forced to keep only the AUC_inf_ as a measure of the total exposure. Obviously, the trapezoidal approximation is closer to the “true” AUC, as the times of the measures are closer. An acritical use of the trapezoidal rule with limited sampling would lead to an incorrect estimate of the AUC, as in the example shown in Supplementary Figure S3. Supplementary Figure S3a compares a complete time course of ASPase activity with frequent sampling (grey circles) vs. a limited sampling, taken with the five-points protocol of the present study (blue circles). The trapezoidal approximation would give in the first case the proper area under the grey line, and in the second case the area under the blue dashed line, heavily underestimated. To mitigate the error, we estimated the AUC as shown in Supplementary Figure S3b (corrected limited sampling, orange circles and line), assuming that the level of the first measured activity after each administration was constant from the end of the 2-h administration to the first measure, one week later. Because the activity is expected to decrease in this period, this correction reduces but does not eliminate the error. In the case shown in Supplementary Figure S3, the uncorrected estimate of the AUC with a limited sampling was 27.2% lower than that with the whole data set (panel a), while with the correction, the area under the orange line was only 9.9% lower (panel b). A similar analysis with the data reported by Rizzari et al. [32] led to reduce the error from 22% to 5%. A better approximation could have been achieved if the peaks of the concentration curve were known (i.e., with a blood sampling immediately after PEG-ASP administrations), but in the absence of data in the first 7 days after each administration, we avoided introducing further arbitrary assumptions and we conservatively adopted the above correction.

Another issue to be considered for the estimate of the AUC concerned the cases where a measure was not made immediately before the second administration. Actually, in 56 (12.9%) instances the second administration was given one or more days after the previous measure. In all these cases the estimate of the AUC required also calculating the area in the interval between the time of the previous measure (t_1_) and the time of the second administration (t_2_). For this purpose, we considered that the interval was within the terminal elimination phase of the first administration, and we assumed that the elimination occurred with the same rate that was measured after the second one. Thus, we estimated the activity at the time of the second administration (A(t_2_)) from the previous measure A(t_1_) with an exponential decrease with rate *k_el_* (A(t_2_) = A(t_1_) × $e^{-k_{el}\times \left(t_{2}-t_{1}\right)}$). Supplementary Figure S4 shows an example of this correction. Together, the two corrections exemplified in Figures S3 and S4 will be referred as the “trapezoidal correction method” in the following.

#### 2.4.3. Non-Compliant Schedules

In our study, the reference schedule (Ref) was exactly applied in 42.2% of the cases, raising to 62.0% when including cases presenting one-day differences from the reference (Ref1) and to 73.3% with two-day differences (Ref2). The remaining included cases with one measure 3 days away from reference (Ref3), missing day 7, day 14 or day 21 measures (m7, m14 and m21 respectively), with delayed second administration (del2nd), with delayed day 28 measure (del28) and other less represented cases. Table 2 reports the whole list of the types of non-compliance observed, with the adopted definitions.

Schedule types Ref1, Ref2, Ref3 included some measure or the second administration shifted, respectively, up to one, two or three days in respect to the reference. In these cases, the estimates of the AUC_inf_ were not expected to be biased, applying the corrections described above with the true times of the measures. The other schedule types required specific considerations in order to consider PK parameters as consistent with those obtained with the reference schedule.

In 10 instances, the measure scheduled at day 28 was delayed more than 3 days (Del28). In three instances the delay was such that the measured activity was <LLOQ, thus *k_el_* could not be measured, while the AUC_inf_ was estimated considering the area after that time to be negligible.

Schedules with more than three days delay of the second PEG-ASP administration (Del2nd, 21 instances) were characterized by a longer period of exposure to the drug. Still, we considered the AUC_inf_ a measure of the total exposure comparable with that of the reference schedules, with the same corrections. In 10/21 instances, an extra blood sampling was made after day 14 before the second administration, enabling an estimate of *k_el_* from two long-term measures also after the first administration, enabling *k_el_* to be obtained in three instances when it was not measurable after the second administration.

In schedules with missing data in respect to reference, we investigated whether and how an estimate of *k_el_* and the AUC could be made. This exercise was also motivated by the perspective of a further reduction of the number of blood samplings for PK monitoring. The blood sampling at day 7, 14 or 21 was occasionally missed in our dataset (types m7, m7Del2nd, m7Del28, m14, m21), affecting the estimate of the AUC but not that of *k_el_*, as calculated with the last two points.

#### 2.4.4. Missing Day 7 Measurement

Supplementary Figure S5a shows how the trapezoidal correction adopted for the five-points time courses would act when missing the day 7 measure in a patient with a Ref schedule type, evidencing that the plain application of the trapezoidal correction method, with extrapolation to day 0 of the day 14 measure, would lead to an underestimation of the AUC in respect to the estimate made with five points.

In order to evaluate the extent and regularity of these underestimations, we compared the calculations of the AUC made in the Ref subgroup of patients, with the recalculation of AUC with four points instead of five, removing day 7, i.e., the AUC without day 7 (AUC_w/o d7_) measures (Supplementary Figure S5b). In the absence of the day 7 measure, the trapezoidal correction method estimates the AUC with an absolute error, in respect to the five-points estimate, of 13% on average and lower than 20% in 167/183 (91%) patients. We found a systematic non negligible error but also that the AUC_w/o d7_ was linearly related to the AUC, with a high determination coefficient (R^2^ = 0.9968).

This prompted us to evaluate a further correction of the AUC_w/o d7_, including a multiplicative factor (MF_m7_), i.e., AUC_w/o d7MF_ = AUC_w/o d7_ x MF_m7_, with MF_m7_ given by the best fit slope of the linear relationship (MF_m7_ = 1.1410). Applying this MF_m7_ model in the group with a reference schedule without day 7 measures, the mean of the absolute error was reduced to less than 5% and the cases with error <20% increased to 180/183 (98%) (Supplementary Figure S6a). Then, the MF_m7_ model was validated in the pooled group of the patients with Ref1, Ref2, Ref3, Del28 and Del2nd schedules, again comparing the five points AUC estimate with that with four points without day 7, obtaining a 5.2% mean error and an error <20% in 184/188 (98%) patients (Supplementary Figure S6b). Thus, the correction with the MF_m7_ model allowed us to estimate the AUC in a non-biased way consistent with the five-points estimate when the day 7 measure was missing. In force of these findings, the model was adopted to estimate the AUC in the patients with m7, m7Del28 and m7Del2nd schedule types and the values were included in the final database of PK parameters.

#### 2.4.5. Missing Day 14 Measurement

Supplementary Figure S7 shows the effect of missing the day 14 measure on the AUC estimate. Figure S7a exemplifies how the trapezoidal correction adopted for the five-points time courses would act when missing the day 14 measure, in the same patient with the Ref schedule type of Figure S5a. A minor underestimation of the AUC was expected, applying the terminal decreasing rate starting from the day 7 for estimating the absent day 14 measure. In fact, with this estimate of the day 14 measure, the mean absolute error was 3.3% with no cases exceeding 14% error in the 183 pts with the Ref schedule (Figure S7b). This was confirmed in the pooled group of the pts with Ref1, Ref2, Ref3, Del28 and Del2nd schedules, where the mean absolute error was 3.7% with no cases exceeding 20% error. Due to the low error found when removing the day 14 measure, the AUC estimate of patients with the m14 schedule was considered consistent with that of the reference, without further corrective factors, and was not excluded from the final database of PK parameters.

#### 2.4.6. Missing Day 21 Measurement

Supplementary Figure S8 shows the effect of missing the day 21 measure on the AUC estimate. In the absence of the day 21 measure, a plain extrapolation of the day 21 measure to the time of the second administration (Supplementary Figure S8a) would lead to an underestimation bias, similarly to the missing day 7 case.

The recalculation of the AUC with four points instead of five, removing the day 21 measures (AUC_w/o d21_) in the patients with the Ref schedule type (Supplementary Figure S8b) led the trapezoidal correction method to estimate the AUC with an error, in respect to the five-points estimate, of 24% on average and lower than 20% in 52/183 (28%) patients. We found a systematic non-negligible error but also that the AUC_w/o d21_ was linearly related with the AUC with a high determination coefficient (R^2^ = 0.9903). Proceeding as in the missing day 7 case, we applied a MFm21 model (AUC_w/o d21MF_ = AUC_w/o d21_ × MF_m21_) multiplying AUC_w/o d21_ by a factor MFm21 given by the best fit slope of the linear relationship (MF_m21_ = 1.2955). With this model the mean error was reduced to 7.1% and the cases with error <20% increased to 174/183 (95%) in the group with the reference schedule (Supplementary Figure S9a). Then, the MFm21 model was validated in the pooled group of the patients with Ref1, Ref2, Ref3, Del28 and Del2nd schedules, again comparing the five points AUC estimate with that with four points without day 21, obtaining an 8.7% mean error and an error <20% in 174/188 (93%) patients (Supplementary Figure S9b). Thus, the correction with the MF_m21_ model allowed us to estimate AUC in a way that is consistent with the five-points estimate when the day 21 measure was missing. In force of these findings, the MF_m21_ model was accepted and adopted to estimate the AUC in the patients with the m21 schedule type.

#### 2.4.7. Other PK Parameters

The estimate of the AUC_inf_ and *k_el_* were used to derive the total Clearance (Cl) and the volume of distribution during the terminal phase (Vz) using the classical formulas: Cl = D/AUC_inf_/BSA and Vz = Cl/*k_el_*/BSA, where D was the total dose (IU) and BSA the body surface area (m^2^). In addition to these PK parameters, we considered other quantities that can be measured with the schedules adopted in this study and are possibly indicative of the efficacy of the drug.

The first measure of activity, here 7 days after the first drug administration (A(7)), gives an immediate detection of cases of partial/complete inactivation. A(7) was measured in 390 patients, with one day tolerance on the blood sampling at day 7.

The activity measured 7 days after the second drug administration (A(7_2nd_), i.e., A(21) in the reference schedule) and the Ratio_d7_ = A(7_2nd_)/A(7), comparing ASPase activity at the same time after first and second administrations, enable the disclosure of a reduction of activity, which may be considered in decisions for the prosecution of the therapy. Ratio_d7_ was measured and included in the final PK database in 343 patients where the two measures were available with one day tolerance in respect to 7 days from the previous drug administration.

T_>30_, T_>100_ and T_>600_ were defined as the periods of exposure above the 30 IU/L, the 100 IU/L and the 600 IU/L thresholds, respectively. They were calculated by extrapolation of the time the threshold was reached after the second administration (t_30_ or t_100_ or t_600_) and subtracting the estimate of the period below the threshold between the two doses if the measured or estimated activity at the time of the second administration was below the threshold. We calculated t_30_, t_100_ and t_600_ in the terminal elimination phase after the second administration, assuming an exponential decrease with *k_el_* rate, either between the last two measures or after the last measure. If the threshold was already reached at the time of the penultimate measure, t_30_ and t_100_ were calculated with exponential interpolation between the penultimate and the previous measure. Then, we estimated the time the threshold is or would have reached following the first administration alone (t1_30,_ t1_100_ and t1_600_), assuming an exponential decrease with *k_el_* rate around the last measure before the second administration. The period below a threshold between the two doses was calculated as the remaining time before the second administration (or zero when t1_30_ (t1_100_ or t1_600_) was reached after the second administration).

In summary, the final PK database included *k_el_*, HL, T_>30_, T_>100_, T_>600,_ the AUC_inf_, CL, Vz, A(7), A(7_2nd_) and Ratio_d7_.

**Figure 1:**
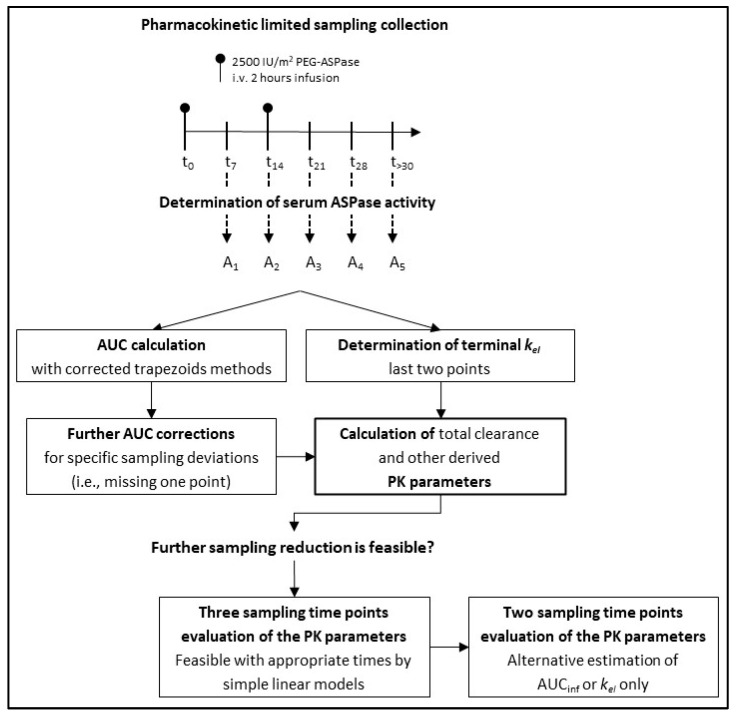
Flowchart of the study.

A flowchart that summarizes our study design is shown in Figure 1.

### 2.5. Statistical Analysis

Frequency distributions of the PK parameters were shown either as cumulative distributions or using box and whiskers plots, where the boxes extend over the interquartile range (IQR), from the first (Q1) to the third (Q3) quartiles; the lines inside the boxes represent the medians, the end of the upper whisker is the largest value within 1.5 × IQR above Q3 and the end of the lower whisker is the smallest value within 1.5 × IQR below Q1. Variability was evaluated with the coefficient of variation (CV), IQR and the range between the 5% and 95% percentiles. Outlier values were defined as higher than Q3 + 1.5 × IQR or lower than Q1 − 1.5 × IQR.

Comparisons between groups were made with an unpaired t test with Welch’s correction, accounting for the unequal variances found (F-test of equality of variances) in most subgroup comparisons. The means were considered significantly different if *p* < 0.05, otherwise the difference was denoted not significant (NS). Statistical significance was specified as *p* < 0.05, *p* < 0.01, *p* < 0.001 and *p* < 0.0001. Limited sampling models of the AUC_inf_ were developed using multivariable linear regression using the Ref subset of patient data and validated with a subgroup comprising all other available patient data. Model accuracy was evaluated using the sum of square errors (SSE) and the Akaike information criterion (AIC).

The percentage of cases predicted (AUC_pred_) that differed less than 10% or 20%, from the measured value (AUC_exp_), with all available time points was also recorded (%Pts_E<10%_ and %Pts_E<20%_).

The performance of the model in the validation subset of patients was evaluated comparing the means of the AUC_exp_ and AUC_pred_, their difference (mean predictive error, as percent of the mean AUC_exp_, MPE%), the percent of root mean square prediction error (RSME%), the mean (E%_mean_) and maximum (E%_max_) of the absolute percent errors in the patients of subset, %Pts_E<10%_ and %Pts_E<20%_.

All analyses were performed using Microsoft Excel 2019 (Microsoft, Redmond, WA, USA) or Prism 10 software (GraphPad Software Inc., La Jolla, CA, USA).

## 3. Results

### 3.1. SAA Levels

**Figure 2:**
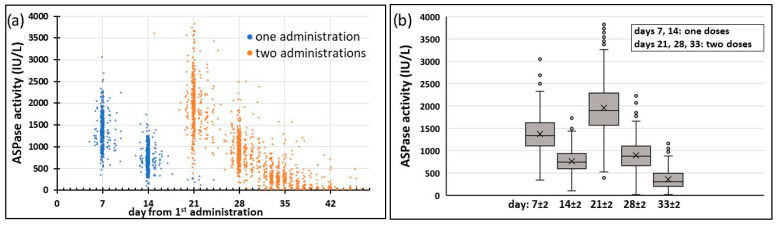
Pooled measures of ASPase activity (IU/L) versus time. (**a**) 2122 individual measures from 434 patients during the induction phase of the protocol. Blue dots represent measures following the first PEG-ASP and orange dots following the second administrations. (**b**) Grouped measures at days 7 (401 pts), 14 (424 pts), 21 (377 pts with two administrations), 28 (375 pts) and 33 (272 pts) with two-days tolerance (1849 measures). Means values are highlighted with a cross in the box and whiskers plot; circles represent individual outlier values (higher than Q3 + 1.5 × IQR or lower than Q1 − 1.5 × IQR).

Figure 2a shows the serum pharmacokinetic profile of ASPase activity analyzed in 2212 serum samples obtained from 434 patients monitored during the induction phase of the study protocol.

Figure 2b shows the box and whisker plot of the measures at days 7, 14, 21, 28 and 33, grouping the measures with two days tolerance. Average ASPase activity was 1380 IU/L 7 ± 2 days after the first dose and 1948 IU/L after the second dose. ASPase activity was still on average at high levels (358 IU/L) on day 33 ± 2, with 90.1% patients maintaining adequate levels above 100 IU/L and only 4.4% of patients with activity below 30 IU/L on that day.

### 3.2. Statistics and Frequency Distribution of PK Parameters

**Figure 3:**
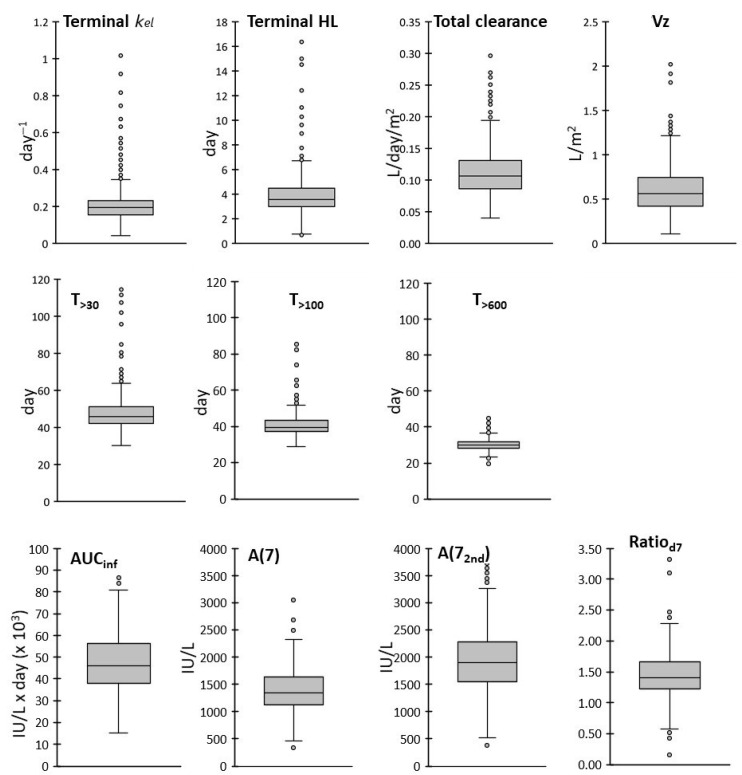
Box and whiskers plot of the distributions of the PK parameters.

Table 3 lists the average value and variability ranges of the PK parameters defined in the methods section. All frequency distributions were superimposable to Gaussian curves between first (Q1) and third (Q3) quartiles, while a deviation from normality with a right tail was observed outside the fitting range with most parameters (Supplementary Figure S10). The outliers shown as circles in the box and whisker plots (Figure 3) highlight the cases with particularly high or low parameter values to be kept under particular attention. In addition, exposure times above thresholds different than 30, 100 or 600 IU/L were considered (Supplementary Table S1).

Terminal *k_el_* distribution was characterized by a relatively narrow IQR and by a right tail, with 27 outlier patients with a fast elimination rate. The terminal HL was 3.92 days on average, and the right tail includes 23 outlier patients with a slow elimination, 14 of them also being outliers for T_>100_ or/and T_>30_. ASPase activity remained higher than 30 IU/L at least for 30 days (minimum T_>30_), with average of 47.9 days, and ASPase activity higher than 100 IU/L was maintained at least 28 days (minimum T_>100_), with average of 41.0 days. The distributions of T_>30,_ T_>100_ and T_>600_ were narrower than those of the other parameters, with CVs of 21.6%, 17.6% and 11.5%, respectively. The AUC_inf_ was distributed as a Gaussian curve with a mean of 46,937 IU/L × day and a 28.5% CV. Total clearance was 0.114 L/day/m^2^ on average, with a distribution skewed on the right side and 17 outlier patients (with accelerated clearance above 0.199 L/day/m^2^), who were not outliers for other parameters. Vz, the volume of distribution based on the terminal slope, was 0.61 L/m^2^ on average with 11 outliers (with Vz above 1.231 L/m^2^), 8 of which were also outliers for HL. A(7) was normally distributed with an average of 1379 IU/L and a 28.2% CV, while A(7_2nd_) had an average of 1946 IU/L. The distribution of A(7_2nd_) was skewed on the right with 12 outliers (above 3385 IU/L), which were not outliers for other parameters beside the related Ratio_d7_. A(7_2nd_) was generally higher than A(7), with a 33% excess indicated by the Ratio_d7_ which was 1.33 on average. This excess was not fully explained by the residual of the first administration expected at this time (day 21 in the reference schedule). An estimate of the contribution of the first dose at day 21, based on the data at day 14 and the terminal *k_el_*, was made in 296 pts with sampling at both days 7 ± 1 and 21 ± 1. Among them, the contribution of the first dose at day 21 was 212 IU/L on average, explaining only about one third of the observed measured average difference between A(7_2nd_) and A(7). Moreover, particularly high activity outlier values of A(7_2nd_) or/and Ratio_d7_ were present in a subgroup of 17 patients. These findings are indicative of a lower decrease in the activity in the first week after the second dose than after the first.

### 3.3. PK Parameters in Patients’ Subgroups

Patients were stratified by sex, age, leukemia immunophenotype (B-cell precursor-, BCP- and T-ALL) and risk group. The frequency distributions of the PK parameters were analyzed in each subgroup.

**Figure 4:**
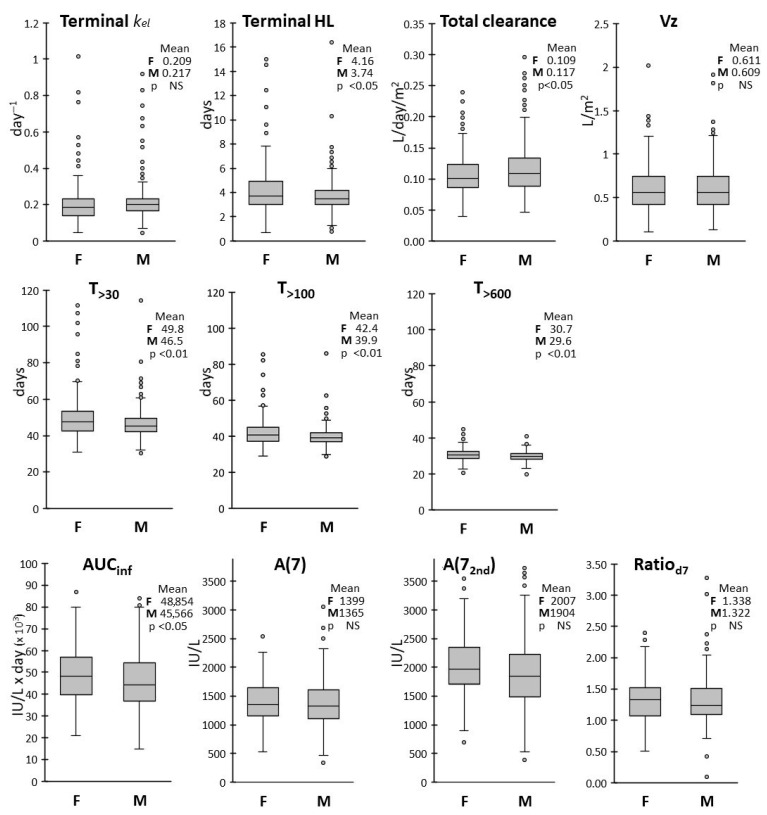
Box and whiskers plots of the distributions of the PK parameters according to sex. Mean values for female (n = 178) and male (n = 248) patients are reported in insets, with corresponding *p*-values from the Welch’s *t* test.

The distributions of all PK parameters were widely overlapping in male and female patients (Figure 4). However, the means of HL, T_>30_, T_>100_, T_>600_ and the AUC_inf_ were significantly higher and the mean of CL lower in females in respect to males, suggesting a trend for higher drug exposure in females.

**Figure 5:**
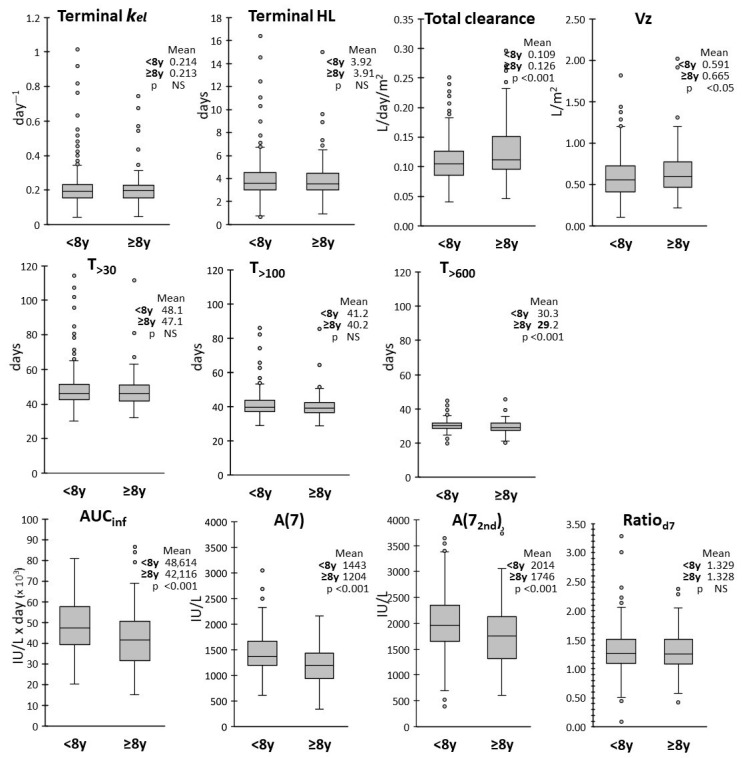
Box and whiskers plots of the distributions of the PK parameters according to age. Mean values for <8 years (n = 285) and ≥8 years (n = 97) patients are reported in insets, with corresponding *p*-values from the Welch’s *t* test.

Patients were stratified according to age as ≥8 years or < 8 years. A higher exposure was found in younger patients in terms of mean AUC_inf_, A(7) and A(7_2nd_), with a lower clearance and Vz (Figure 5).

**Figure 6:**
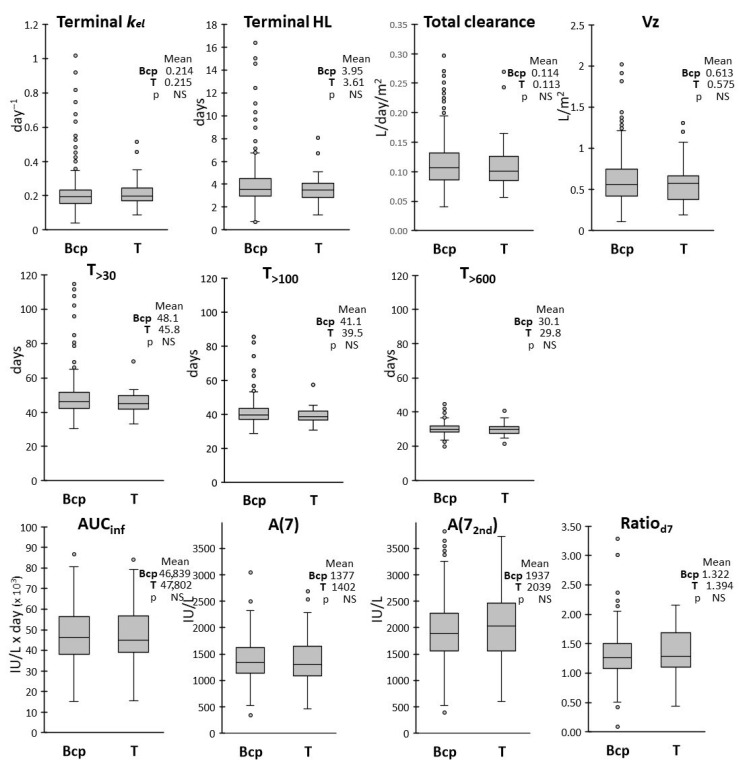
Box and whiskers plots of the distributions of the PK parameters according to leukemia type. Mean values for BCP- (n = 385) and T-ALL (n = 41) patients are reported in insets, with corresponding *p*-values from the Welch’s *t* test.

Leukemia immunophenotype did not influence any PK parameters, with a complete overlapping of the distribution between BCP- and T-ALL patients (Figure 6).

**Figure 7:**
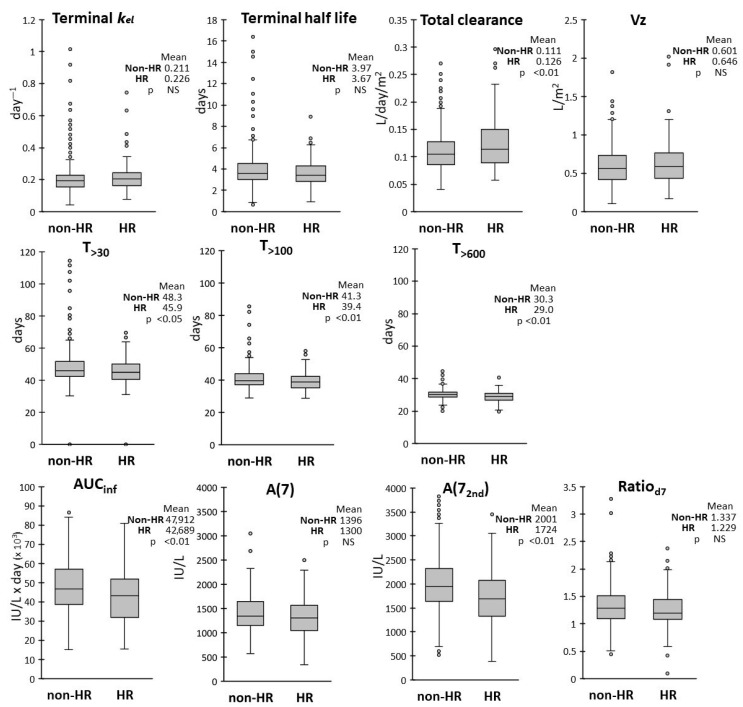
Box and whiskers plots of the distributions of the PK parameters according to risk level, high (HR, n = 321) or not (non-HR, n = 69). Mean values for non-HR and HR patients are reported in insets, with corresponding *p*-values from the Welch’s *t* test.

In respect of the risk group categories, patients were stratified as high risk (HR) or non-HR. The HR group was associated with a lower AUC_inf_ and A(7_2nd_), but not A(7), suggesting that the reduction of exposure occurred after the second administration (Figure 7).

### 3.4. Further Reductions of the Number of Measures

The goal of any therapeutic monitoring is the maximum reduction of the blood sample numbers. We showed above that the reference five-measures protocol could be reduced to four, skipping either day 7, day 14 or day 21 measurements, maintaining the estimate of terminal *k_el_* and derivate quantities with the last two measures, on day 28 and later, and by applying simple corrective multiplicative factors for the estimate of the AUC_inf_. We also evaluated the performance of other linear models for estimation of the AUC_inf_, based on three or less measures. Several linear combinations of three measures were considered, obtaining the best fit parameter values in the subset of patients with the Ref schedule type (training set). The AUC predicted by the model (AUC_pred_) with limited sampling was compared with the AUC_inf_ (AUC_exp_) and the relative absolute error, as percent of the AUC_exp_ (%E), was calculated for each patient. Table 4 reports the formulas, best fit parameter values (c_0_, c_1_, c_2_ and c_3_), the value of the objective function (sum of square errors, SSE), minimized in the training set, the Akaike Information Criterion (AIC) adopted to compare models with a different number of parameters and the percentage of patients with absolute error lower than 10% (%Pts_E<10%_) and 20% (%Pts_E<20%_). A model without intercept (c_0_) was adopted when leading to a lower AIC.

Each model was validated in the subset of the other patients with measures available at the sampling days specified by the model, with 2 days tolerance (validation set). Table 5 reports the performance of the model in the validation subset of patients, including the means of the AUC_exp_ and AUC_pred_, their difference (mean predictive error, as percent of the mean AUC_exp_, MPE%), the percent of root mean square prediction error (RSME%), the mean (E%_mean%_) and maximum (E%_max_) of the absolute percent errors in the patients of subset, %Pts_E<10%_ and %Pts_E<20%_.

#### 3.4.1. Models with Three Sampling Time Points

Mod A, with sampling days 7, 21 and 28 reached the lowest AIC among the tested models, with 5.0% mean absolute error, and only one patient with an error exceeding 20% in the training and validation sets. Considering the similar best fit values of the three parameters, we tested a simpler one-parameter model with the sum of the three measures (Asum); Mod Asum actually performed as well as mod A, with a lower AIC, demonstrating that the AUC_inf_ was simply proportional to the sum of the measures at days 7, 21 and 28.

Mod B and F are similar to mod A, with the two measures 7 days after each administration but with day 14 (B) or after day 30 (F) as the third measure. Both models performed only slightly worse than mod A, with %Pts_E<20%_ >98% in both subsets, but with a lower %Pts_E<10%_. The maximum error was 39.1% in mod B.

Mod E uses only the measures after the second administration. It performs worse than mods B and F, with the mean error rising to 9.9% and %Pts_E<10%_ reducing to 62.4, but still %Pts_E<20%_ was near 90% (89.5%) in the validation set.

Mod G was similar to mod E, using the last two measures at days 28 and after day 30, but keeping day 7 instead of day 21. The model fitted worse than mod E in the training set, with a higher SSE and AIC, but performed similarly in the validation set. Both mods E and G allow estimation of *k_el_* with day 28 and day >30 measures.

#### 3.4.2. Models with Two Sampling Time Points

Starting from the best three-measures mods A and Asum, we considered the simpler mod C, with only two samplings at day 7 after each administration. Mod C actually performed quite similarly to mod B, and it was the best two-samplings model for the AUC, also in the Csum variant.

Mods D and H were derived from mods E and G, respectively, skipping the last measure. Their performance was almost identical to that of the three-samplings models, indicating that the last measure was not necessary for the estimate of the AUC_inf_. In both cases, the models were successfully simplified to the Dsum and Hsum variants, considering the sum of the two measures and reducing the number of parameters.

Thus, it is possible to estimate the AUC_inf_ with two-points models. However, the times required for these models do not allow for estimation of *k_el_* and derived PK parameters, like t_>100_, for which longer time points are required.

#### 3.4.3. Models with One Sampling Time Point

We then evaluated the capability of single-measure models to predict the AUC, from a single measure 7 days after the first (mod A(7)) or after the second (mod A(21)) administration. We found that mod A(7) was inadequate, as it estimated the AUC with a mean error of 18.3%, with about one third of the patients with an error higher than 20%, up to a maximum above 100%. Instead, the performance of mod A(21) might be acceptable, predicting the AUC with an error of 11.4% on average, lower than 20% in 83.7% of patients and with a maximum error of 52%.

Taken together, these results suggest that two-samplings models are sufficient to estimate with high accuracy the AUC (with mod D_(21,28)_ or mod H_(7,28)_) or *k_el_* (with the last two measures at A(28) and A(>30), as shown in Equation (1)), but not both. At least three samplings are necessary for a complete PK characterization, as in mod E_(21,28,>30)_ and mod G_(7,28,>30)_, which represents a valid trade-off for reducing sampling number while maintaining high informative content.

## 4. Discussion

In this paper, we reported the results of a pharmacokinetic study based on the measurement of SAA in a very large cohort of children and adolescents affected by ALL and treated with PEG-ASPase in the induction phase. The study was carried out within the AIEOP-BFM ALL2009 study, which included an extensive use of the PEG-ASPase product and SAA monitoring during the treatment. The main objective of this monitoring plan was to evaluate the profile of the SAA and to evaluate the incidence of the inactivation of PEG-ASPase.

SAA measurements are usually performed one and/or two weeks after any PEG-ASPase dose administration. Low or absent SAA levels are often accompanied by the presence of anti-ASPase antibodies, which, in such cases, oblige a switch to a different formulation of ASPase, i.e., the *E. chrysanthemi* product [6]. There is a broad consensus that an adequate ASN depletion can be consistently reached when the SAA is above the threshold level of 100 IU/L, at least until the next dose is administered [12,17]. Our results demonstrate that the administration of the two PEG-ASPase doses during the induction phase ensure such therapeutic SAA levels until day 33 in 90.1% of the population investigated, and above 30 IU/L in 95.6% of the population, having been reported that 30 IU/L threshold is likely sufficient to obtain an effective Asn depletion in a high percentage of patients [17,19].

The profile of the SAA values here reported is in keeping with those published by Würthwein et al. [24], and found within the same AIEOP-BFM ALL 2009 study but with patients enrolled in countries other than Italy.

In principle, sequential SAA measurements can be also used to calculate relevant PK parameters, such as the AUC, the clearance or the time when the drug concentration is maintained above given thresholds, which are potentially useful for individual dose refinements of subsequent PEG-ASPase administrations. Moreover, whether the research would demonstrate relevant variations of these parameters in clinically identifiable (by sex, age, leukemia type or other) subgroups of patients, the point of dose/schedule adjustments in those subgroups would be considered in future protocols. On the other hand, current clinical practice is oriented to reduce as much as possible blood sampling for SAA measures.

The AIEOP-BFM ALL 2009 protocol included two PEG-ASPase doses 14 days apart and five serum samplings to measure PEG-ASPase activity over more than 30 days. In this limited-sampling plan, the evaluation of the PK parameters could be difficult and would imply methodologically and interpretably complex aspects that have been previously tackled by means of population pharmacokinetic modeling [13,24,25,26,27,28]. In such studies, structural compartmental models were developed taking into account the time-dependence of the clearance of PEG-ASPase, mainly due to progressive de-pegylation of the PEG-ASPase molecules.

In our study we have demonstrated that a direct estimation of the main PK parameters in individual patients is possible by using the available and reduced SAA results by adopting simple adjustments to the classical formulae of non-compartmental PK analysis. We also obtained proper estimates of the AUC_inf_ with four samplings, when measures at days 7, 14 or 21 were missing. The two long term measures, at least 2 weeks after the second and last PEG-ASP dose, had to be maintained to estimate the terminal elimination rate, obviously renouncing to evaluate the previous variation of this parameter, but still getting an estimate of the volume of distribution during terminal phase and of the total clearance, considering the two doses. Based on these procedures, we built a database including 434 patients where *k_el_*, terminal HL, T_>30_, T_>100_, T_>600,_ AUC_inf_, CL, Vz, A(7), A(7_2nd_) and Ratio_d7_ could be measured. The average values of the PK parameters and their ranges were consistent with those previously reported for PEG-ASP, taking into account the different derivations. In particular, compartmental models were based on the clearance and estimates of its time-variation, while in our study the total clearance was derived from the total dose and from the estimate of the AUC_inf_. Our average CL estimate (0.114 L/day/m^2^) was close to the initial clearance (0.126 L/day/m^2^) of the compartmental model previously reported by Würthwein et al. [24].

We also found that T_>600_ and T_>100_ were the least variable parameters, with CVs lower than 20%; low variability was found also for T_>30_ (21%), while *k_el_*, terminal HL and Vz showed the highest CVs (around 50%). The frequency distributions of the AUC_inf_ and A(7) were superimposable to normal distributions, while subgroups of patients with higher outlier values were detected in the distributions of some PK parameters, particularly in those of *k_el_* and terminal HL.

We also evaluated whether age, sex, ALL subtype and risk group assignment could affect the drug pharmacokinetics by analyzing the frequency distributions of the PK parameters in each subgroup. ALL subtypes (BCP vs. T-ALL) had no effect on any PK parameters, while modest differences were observed between subgroups with different biological variables.

In respect to sex, the average terminal HL, T_>30_, T_>100_ and T_>600_ were about 10% lower in male vs. female patients, with higher variability observed in the female group. The AUC_inf_ was also slightly lower, with correspondingly higher CL, in male patients, while frequency distributions in the two groups were overlapping for the other PK parameters. Lower clearance in the female group was in keeping with the findings of Würthwein et al. [24].

Age somewhat affected the exposure, with the AUC_inf_, A(7) and A(7_2nd_) about 15% lower and the CL higher in ≥8year-old patients vs. those <8 years-old. An increase of clearance in older patients also confirms the observation already made by Würthwein et al. [24].

We also found a somewhat reduced exposure (T_>30_, T_>100_, T_>600_, AUC_inf_) in HR patients when compared with the non-HR group. However only A(7_2nd_) and not A(7) were reduced, suggesting an effect occurring after the second dose. Clearance was consequently increased by 10% in HR patients, as was also found by Liu et al. [25] in an US cohort treated with a different protocol.

However, despite statistically significant differences of the mean values, the range of observed values in sex, age and risk subgroups were broadly overlapping. Thus, the observed 10–15% differences on the average values seem to be indicative of a trend but are not sufficient for advising an adjustment of dose or schedule in these subgroups.

The value of PK monitoring to control the activity levels above the threshold associated with adequate ASN depletion is commonly accepted [13,25,26,34], but the additional measurements of the main PK parameters would provide additional useful information. Patients with a low AUC_inf_ or high clearance or who experience a variation of the PK parameters may receive particular attention and more frequent PK monitoring to face a reduced exposure to PEG-ASP or the initial signs of drug inactivation. However, the complex clinical practice underlying the treatment of such patients comprehensibly leads to a reduction of the number of samplings, thus making a proper measure of these parameters difficult. We explored the minimal requirements for an estimate of the AUC_inf_ (and thus also clearance) with the database of the cohort of patients of this study. We found that the day 14 measurement is not essential and could be skipped, leading to an otherwise complete PK characterization with all ten PK parameters considered in this study. To obtain a further sampling reduction, we devised and validated simple linear models to estimate the AUC_inf_ in function of the activities measured at three times or less. We found that the AUC_inf_ is related to the sum of three measures at days 7, 21 and 28 by a simple multiplicative factor (mod Asum, {11.2 × [A(7)] + A(21) + A(28)]}. This was the best AUC_inf_ model based on three measures, but the same measures did not allow for estimation of *k_el_* (and thus also of terminal HL, T_>30_, T_>100_), which required at least two long term points. The estimate of almost all PK parameters was achieved with mod E {6316 + [14.3 × A(21)] + [14.1 × A(28)] + [0.6 × A(>30)]} and with mod G {4512 + [17.6 × A(7)] + [21.4 × A(28)] + [0.4 × A(>30)]}. With these models the error was below 20% in 90% of the cases of the validation set, with maximum error of 40%. The measures on days 7 and 21 provided the best two points model (mod C, {[13.3 × A(7)] + [ 14.7 × A(21)]}). Although *k_el_* could not be measured with this schedule, mod C gave a surprisingly good estimate of the AUC_inf_, with only two cases of error above 20% in both the training and validation set. Mod C became even more simple in the Csum variant {14.1 × [A(7)] + A(21)]}, demonstrating that a simple scale factor connects the sum A(7) + A(21) to the AUC_inf_. Instead, the attempt to estimate the AUC_inf_ by using a single point, either at day 7 or at day 21, led to the unsatisfactory Mod A(7) and Mod A(21).

## 5. Conclusions

In conclusion, this study demonstrated that the adoption of simple models can allow the reduction of the sampling schedule from the five-measures used in this cohort of patients to a three-measures protocol, maintaining a full PK characterization. When the main aim is to predict the time above 100 IU/L, or any other threshold, accepting to renounce to an estimate of the AUC_inf_, a further sampling reduction would be feasible, as two long term measures are required to estimate *k_el_* and its derived parameters, according the formula *k_el_* = − ln [A(>30)/A(28)]/[A(>30) − A(28)]. On the contrary, when the main aim is the estimation of AUC and its derived parameters, like clearance, also renouncing to estimate *k_el_*, it can be achieved with the two samplings schedule on days 7 and 21, with mod C_sum_ {14.1 × [A(7)] + A(21)]}, which represents a valid trade-off for reducing sampling number while maintaining a high informative content, allowing also to monitor the activity 7 days after each administration and their ratio.

## Figures and Tables

**Table 1:** Demographic details of the 434 patients (female 41.7%, male 58.3%) entered in the study.

Baseline Characteristics	Median	5–95% Range
Age (years)	4	1–15
Height (cm)	110	85–167
Body weight (kg)	19	11–58
Body mass index (kg/m^2^)	16.2	13.6–21.9
Body surface area (m^2^)	0.76	0.5–1.6
PEG-ASP absolute dose (IU)	1900	1250–3750
PEG-ASP dose (IU/m^2^)	2500	2201–2540
PEG-ASP dose (IU/kg)	100	64.2–115.4

**Table 2:** Definition of the types of actual schedules and their frequency in the dataset.

**Ref (reference):**	second administration at day 14, five measures at days 7/14/21/28/ > 30	**183 pts**	**(42.2%)**
**Ref1:**	second administration and/or some measure differing one day from the reference (with all other measures as in the Ref schedule)	**86 pts**	**(19.8%)**
**Ref2:**	second administration and/or some measure differing two days from the reference (with criteria Ref or Ref1 met for all other measures)	**49 pts**	**(11.3%)**
**Ref3:**	second administration or one measure differing three days from the reference (with criteria Ref, Ref1 or Ref2 met for all other measures)	**30 pts**	**(6.9%)**
**m7:**	missing day 7, the other four measures met Ref, Ref1 or Ref2 criteria	**23 pts**	**(5.3%)**
**m14:**	missing day 14, only one measure at day 7 before the second administration	**9 pts**	**(2.1%)**
**m21:**	missing day 21	**17 pts**	**(3.9%)**
**Del2nd:**	delayed second administration (>17 days after the first one) (with the following measures shifted accordingly)	**21 pts**	**(4.8%)**
**Del28:**	measure scheduled at day 28 delayed more than three days (with criteria Ref or Ref1 met for all other measures)	**10 pts**	**(2.3%)**
**m7Del2nd:**	missing day 7 and delayed second administration	**2 pts**	**(0.5%)**
**m7Del28:**	missing day 7 and day 28 delayed	**4 pts**	**(0.9%)**
	**TOT**	**434 pts**	**(100%)**

**Table 3:** Average values and variability range of the PK parameters. A(7) and A(7_2nd_) measures were accepted with one day tolerance.

PK Parameter	Unit	N	Mean	CV%	Median	IQR	5–95% Range
**Terminal *k_el_***	day^−1^	426	0.214	54.3	0.194	0.078	0.101–0.431
**Terminal HL**	day	426	3.92	46.1	3.56	1.51	1.61–6.87
**T_>30_**	day	426	47.9	21.6	45.8	9.1	35.2–65.6
**T_>100_**	day	426	41.0	17.6	39.6	6.2	32.1–53.0
**T_>600_**	day	416	30.1	11.5	30.0	3.4	24.7–35.6
**AUC_inf_**	IU/L × day	434	46,937	28.5	46,131	18,488	25,081–69,244
**Cl**	L/day/m^2^	434	0.114	34.0	0.106	0.045	0.068–0.188
**Vz**	L/m^2^	426	0.61	44.9	0.56	0.32	0.25–1.11
**A(7)**	IU/L	390	1379	28.2	1341	512	778–2072
**A(7_2nd_)**	IU/L	382	1946	31.2	1896	733	979–3056
**Ratio_d7_**	-	343	1.47	26.5	1.41	0.44	0.92–2.13

**Table 4:** Models with three or less samplings. Training set (Ref schedule, 183 patients).

Model (Sampling Days)	Formula	Nr. Sample	Nr. Par.	C_0_	C_1_	C_2_	C_3_	SSE × 10^9^	AIC	%Pts_E<10%_	%Pts_E<20%_
Mod A_(7,21,28)_	**c_1_∙A(7)+c_2_∙A(21)+c_3_∙A(28)**	3	3		11.0	11.0	11.8	1.49	2921	93.4%	99.5%
Mod Asum_(7,21,28)_	**c_1_∙(A(7)+A(21)+A(28))**	3	1		11.2			1.49	2916	94.0%	99.5%
Mod B_(7,14,21)_	**c_1_∙A(7)+c_2_∙A(14)+c_3_∙A(21)**	3	3		9.7	9.2	13.6	2.81	3037	84.7%	99.5%
Mod F_(7,21,>30)_	**c_1_∙A(7)+c_2_∙A(21)+c_3_∙A(>30)**	3	3		13.0	14.1	7.3	2.72	3031	84.2%	98.9%
Mod E_(21,28,>30)_	**c_0_+c_1_∙A(21)+c_2_∙A(28)+c_3_∙A(>30)**	3	4	6316	14.3	14.1	0.6	3.74	3092	74.9%	96.7%
Mod G_(7,28,>30)_	**c_0_+c_1_∙A(7)+c_2_∙A(28)+c_3_∙A(>30)**	3	4	4512	17.6	21.4	0.4	5.81	3173	72.7%	90.7%
Mod C_(7,21)_	**c_1_∙A(7)+c_2_∙A(21)**	2	2		13.3	14.7		3.19	3057	79.8%	98.9%
Mod Csum_(7,21)_	**c_1_∙(A(7)+A(21))**	2	1		14.1			3.20	3055	80.9%	98.9%
Mod D_(21,28)_	**c_0_+c_1_∙A(21)+c_2_∙A(28)**	2	3	6355	14.3	14.3		3.74	3089	74.3%	96.7%
Mod Dsum_(21,28)_	**c_0_+c_1_∙(A(21)+ A(28))**	2	2	6353	14.3			3.74	3086	74.3%	96.7%
Mod H_(7,28)_	**c_0_+c_1_∙A(7)+c_2_∙A(28)**	2	3	4554	17.5	21.5		5.81	3170	71.6%	90.7%
Mod Hsum_(7,28)_	**c_0_+c_1_∙(A(7)+ A(28))**	2	2	4003	19.3			5.91	3170	56.8%	88.5%
Mod A_(7)_	**c_0_+c_1_∙A(7)**	1	2	12,127	26.5			13.29	3318	43.2%	78.1%
Mod A_(21)_	**c_0_+c_1_∙A(21)**	1	2	9444	19.2			6.27	3181	64.5%	94.0%

**Table 5:** Models with three or less samplings. Validation set (patients with a non-Ref schedule and available measures of the model, with two days tolerance).

Model (Sampling Days ± 2)	Nr. Pts	Mean AUC_exp_	Mean AUC_pred_	MPE%	RSME%	E%mean	E%max	%Pts_E<10%_	Pts_E<20%_
Mod A_(7,21,28)_	133	44,952	45,033	0.18%	6.9%	5.0%	26.6%	88.7%	99.2%
Mod Asum_(7,21,28)_	133	44,952	45,055	0.23%	6.9%	4.9%	27.1%	88.0%	99.2%
Mod B_(7,14,21)_	154	44,387	44,547	0.36%	8.3%	5.7%	39.1%	81.2%	98.1%
Mod F_(7,21,>30)_	146	45,077	45,761	1.52%	7.0%	5.3%	26.5%	87.0%	97.9%
Mod E_(21,28,>30)_	133	45,325	45,049	−0.61%	12.3%	9.9%	39.0%	62.4%	89.5%
Mod G_(7,28,>30)_	131	44,952	45,615	2.47%	10.1%	8.5%	33.4%	66.4%	93.1%
Mod C_(7,21)_	155	44,449	44,617	0.38%	8.3%	6.2%	35.6%	80.0%	98.7%
Mod Csum_(7,21)_	155	44,449	44,714	0.60%	8.4%	6.3%	35.3%	78.1%	97.4%
Mod D_(21,28)_	135	45,227	44,989	−0.53%	12.4%	10.0%	38.8%	63.0%	89.6%
Mod Dsum_(21,28)_	135	45,227	44,989	−0.53%	12.4%	10.0%	38.8%	63.0%	89.6%
Mod H_(7,28)_	155	44,449	44,682	0.53%	11.5%	9.8%	37.1%	61.3%	87.1%
Mod Hsum_(7,28)_	155	44,449	40,881	−8.03%	13.8%	11.2%	46.2%	52.9%	84.5%
Mod A_(7)_	218	45,065	47,517	5.44%	20.1%	18.3%	121.9%	39.4%	68.3%
Mod A_(21)_	153	44,975	45,025	0.11%	13.7%	11.4%	52.0%	52.3%	83.7%

## Data Availability

The datasets generated and/or analyzed during the current study are not publicly available due to data protection rules and the fact that the complexity of the data does not allow for full anonymization.

## Supplementary Materials

The following supporting information can be downloaded at: https://www.mdpi.com/article/10.3390/pharmaceutics17070915/s1, Figure S1. Flow chart of each patient’s selection for pharmacokinetic analysis. Figure S2. Comparison of options for estimating *k_el_* in 345 cases with the last measure >LLOQ. Figure S3. Example of the AUC correction with a limited sampling strategy. Figure S4. The AUC correction in cases where the last measure before the second administration was not on the same day. Figure S5. The effect of missing the day 7 measure on the AUC estimate. Figure S6. Performance of the MF_m7_ model. Figure S7. The effect of missing the day 14 measure on the AUC estimate. Figure S8. The effect of missing the day 21 measure on the AUC estimate. Figure S9. Performance of the MF_m21_ model. Figure S10. Cumulative distribution of the experimental data (exp) and of the gaussian curve fitting the data between *p* = 0.25 (Q1) and *p* = 0.75 (Q3). Table S1. Average values and variability range of the exposure times above the specific activity thresholds.
