# Supplementary material for: Pharmacokinetics of Pegaspargase with a Limited Sampling Strategy for Asparaginase Activity Monitoring in Children with Acute Lymphoblastic Leukemia
Source: Pharmaceutics. 2025 Jul 15;17(7):915. doi: 10.3390/pharmaceutics17070915 (PMC12297903; doi:10.3390/pharmaceutics17070915)
Supplement: Supplementary file 1 [file pharmaceutics-17-00915-s001.zip › pharmaceutics-3602010-supplementary.pdf]

Pharmacokinetics of Pegaspargase with a Limited Sampling Strategy for Asparaginase Activity Monitoring in Children with Acute Lymphoblastic Leukemia

Cristina Matteo [1,†], Antonella Colombini [2,†], Marta Cancelliere [1], Tommaso Ceruti [1], Ilaria Fuso Nerini [1,‡], Luca Porcu [1], Massimo Zucchetti [1,*], Daniela Silvestri [3], Maria Grazia Valsecchi [4,5], Rosanna Parasole [6], Luciana Vinti [7], Nicoletta Bertorello [8], Daniela Onofrillo [9], Massimo Provenzi [10], Elena Chiocca [11], Luca Lo Nigro [12], Laura Rachele Bettini [2,13], Giacomo Gotti [2], Silvia Bungaro [2], Martin Schrappe [14], Paolo Ubezio [1,§] and Carmelo Rizzari [2,§]

Supplementary information

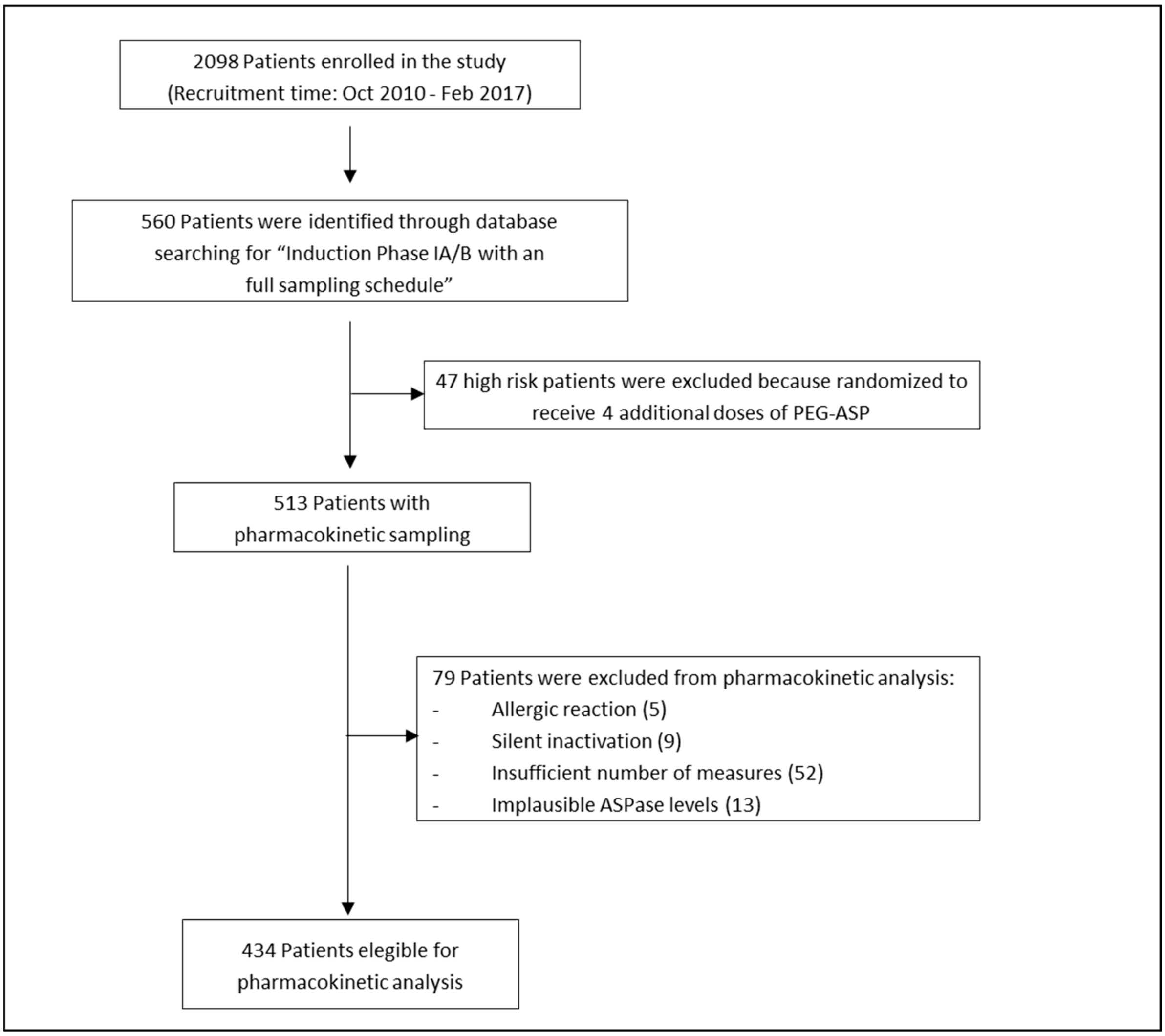

Figure S1. Flow chart of patient's selection for pharmacokinetic analysis.

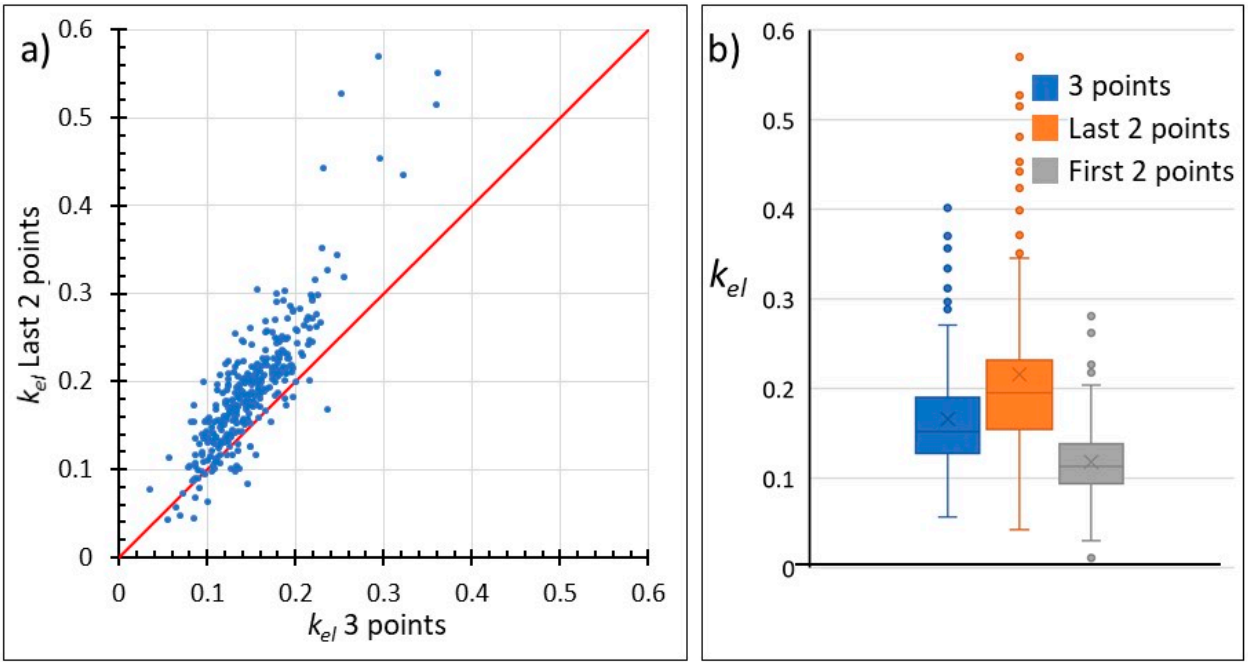

Figure S2. Comparison of options 1 (k_{el} 3 points) and 2 (k_{el} last 2 points) for estimating k_{el} in 345 cases with last measure >LLOQ (**panel a**) Box and whiskers plot of the distributions of k_{el} estimated with options 1, 2 and 3 (k_{el} first 2 points) in all cases allowing the use of each option (**panel b**). The cross in each box indicates the mean value.

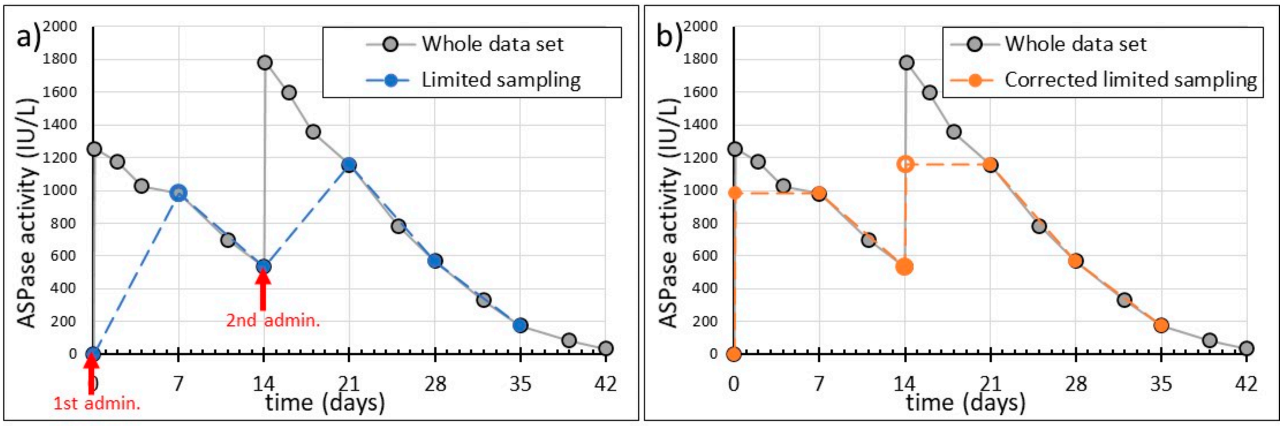

Figure S3. Example of AUC correction with limited sampling strategy.

The data of a complete time course are derived from Schore et al. [17] and represents the means of activity measures (15 time points) in 86 patients after a single PEG-ASP administration and replicated for the 2nd dose at day 14 (grey symbols with continuous line), adding thereafter to the residual activity of the 1st dose. In case of limited sampling with the 5 time points of the reference protocol adopted in the present AIEOP trial, the AUC calculated with the trapezoidal rule without correction would be the area under the blue dashed line, underestimating by 27.2% the “true” AUC, under the grey line (**panel a**). The adopted correction led to estimate AUC with the area under the dashed orange line (**panel b**), reducing the underestimation to 9.9%.

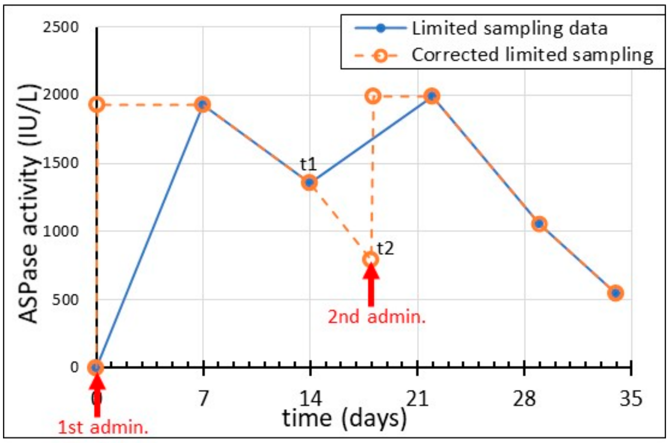

Figure S4. AUC correction in cases where the last measure before the 2nd administration was not in the same day. In the example the 2nd administration was given at day 18 and the previous measure was at day 14. The decrease of activity between days 14 and 18 was estimated from the day 14 measure assuming exponential decrease with rate k_{el} measured in the terminal elimination phase at the end of the time course.

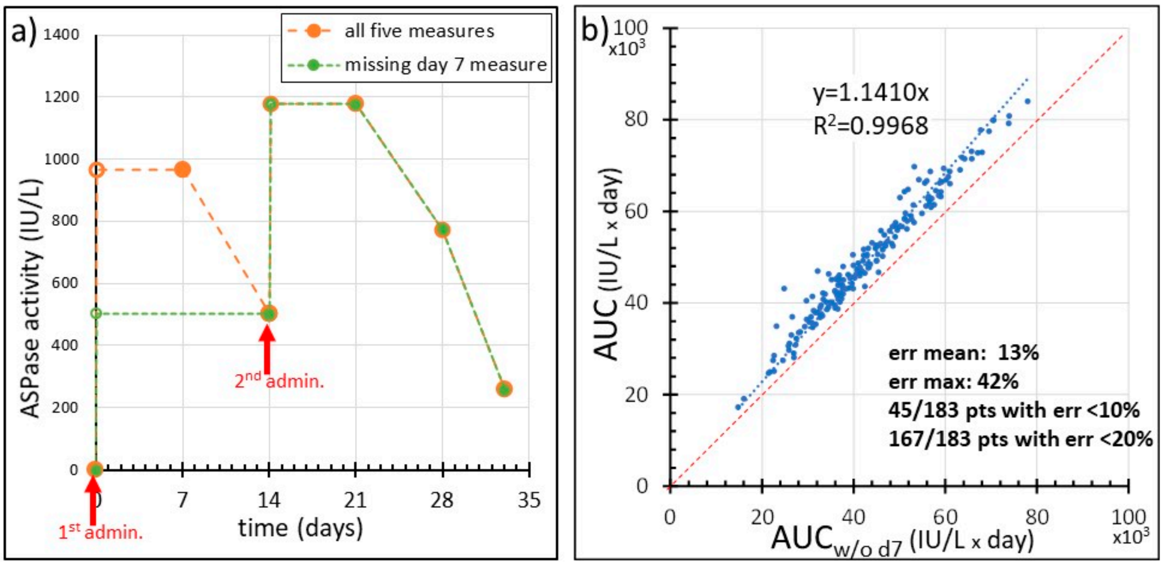

Figure S5. Effect of missing day 7 measure on AUC estimate.

Panel a: typical five-measures PK profile with the trapezoidal correction (orange) compared with the four-measure resulting PK profile applying the same correction method when missing day 7 measure. **Panel b**: Comparison of the estimates of AUC_{inf} with the trapezoidal correction in the group of patients with reference schedule (*y* axis), with the estimate made in the same patients without the measure at day 7 (*x* axis). The dotted blue line shows the best fit linear regression connecting the two estimates, with the equation and R^2 reported in the panel. The red line at 45° highlights the exact correspondence between the two estimates.

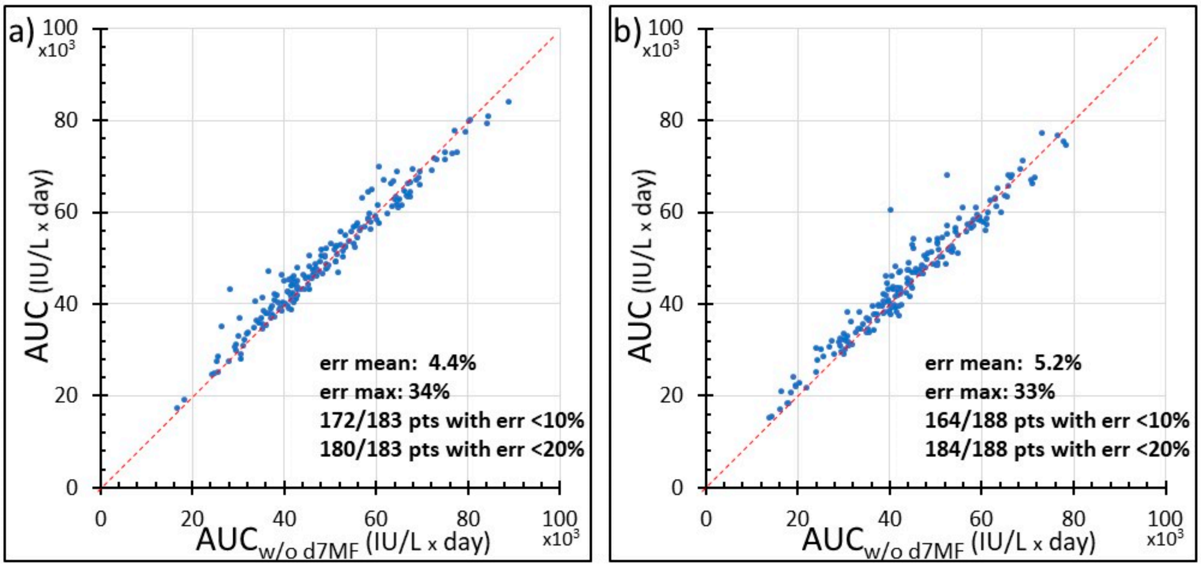

Figure S6. Performance of the MF_{m7} model.

Comparison of the estimates of AUCinf with five points (*y* axis), with the estimates made in the same patients without the measure at day 7 applying the MF_{m7} model (*x* axis). **Panel a**: patients with the reference schedule; **panel b**: pts with Ref1, Ref2, Ref3, Del28, Del2nd schedules. The red line at 45° highlights the exact correspondence between the two estimates.

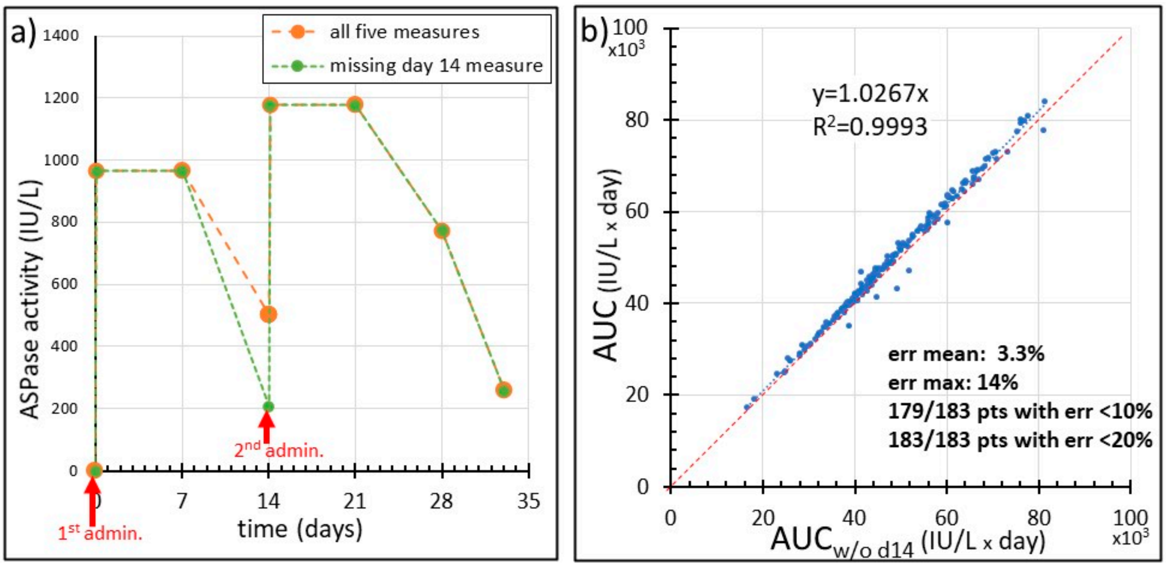

Figure S7. Effect of missing day 14 measure on AUC estimate.

Panel a: typical five-measures PK profile with the trapezoidal correction (orange) compared with the four-measure PK profile with estimation of the missing day 14 applying the terminal decreasing rate starting from day 7. **Panel b**: Comparison of the estimates of AUC_{inf} with the trapezoidal correction in the group of patients with reference schedule (*y* axis), with the estimate made in the same patients without the measure at day 14 (*x* axis). The dotted blue line shows the best fit linear regression connecting the two estimates, with the equation and R^2 reported in the panel. The red line at 45° highlights the exact correspondence between the two estimates.

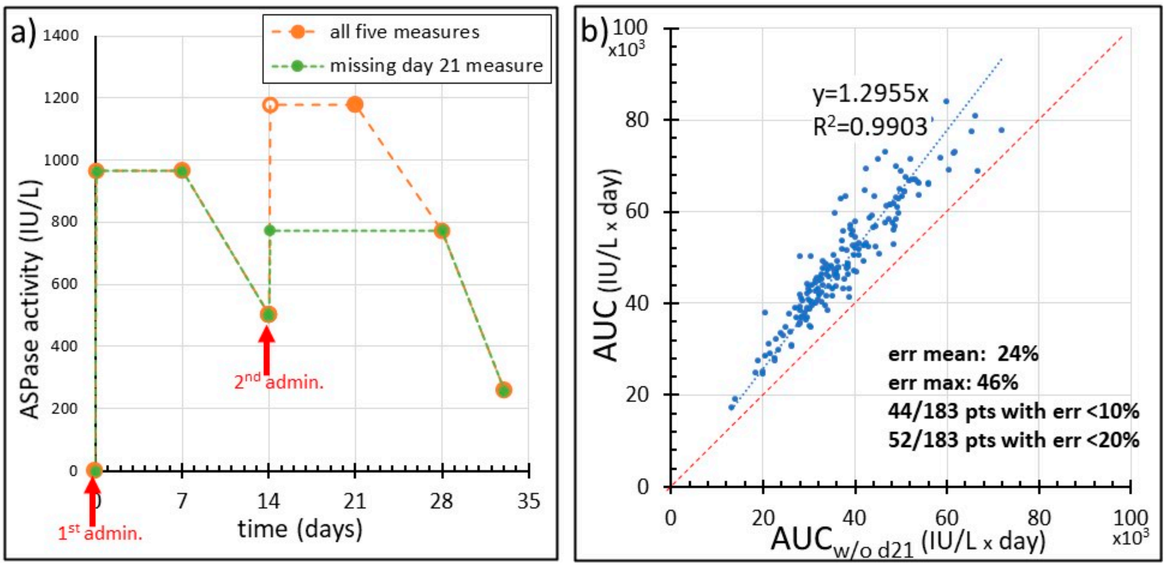

Figure S8. Effect of missing day 21 measure on AUC estimate.

Panel a: typical five-measures PK profile with the trapezoidal correction (orange) compared with the four-measure resulting PK profile applying the same correction method when missing day 21 measure. **Panel b**: Comparison of the estimates of AUC_{inf} with the trapezoidal correction in the group of patients with reference schedule (*y* axis), with the estimate made in the same patients without the measure at day 21 (*x* axis). The dotted blue line shows the best fit linear regression connecting the two estimates, with the equation and R^2 reported in the panel. The red line at 45° highlights the exact correspondence between the two estimates.

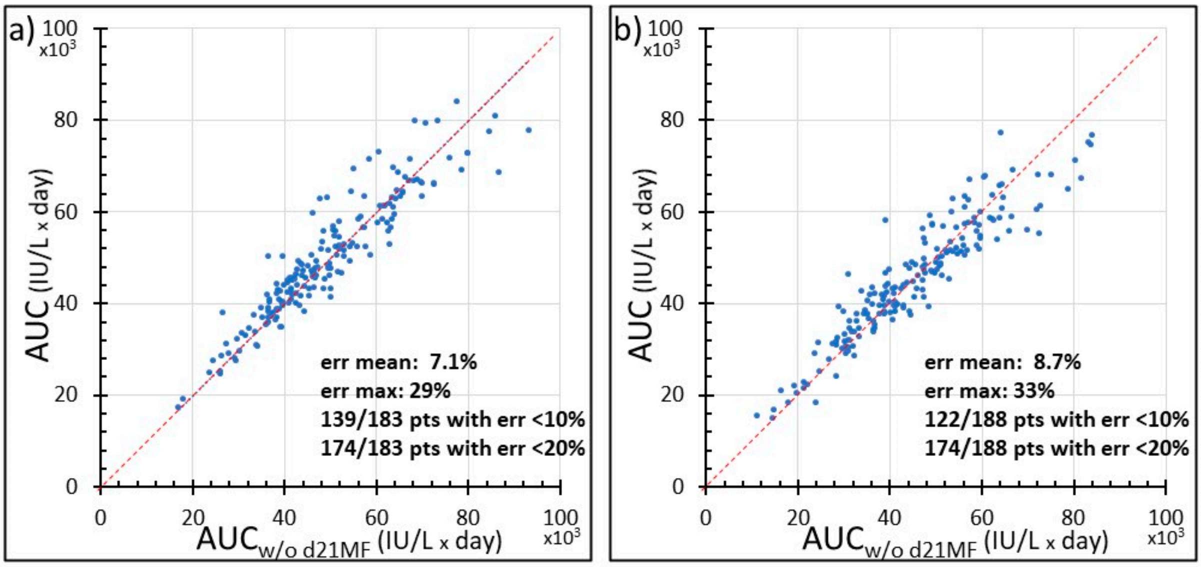

Figure S9. Performance of the MF_{m21} model.

Comparison of the estimates of AUC_{inf} with the trapezoidal correction with five points (*y* axis), with the estimate made in the same patients without the measure at day 21 with correction with the MF_{m21} model (*x* axis). **Panel a**: patients with the reference schedule; **panel b**: patients with Ref1, Ref2, Ref3, Del28, Del2nd schedules. The red line at 45° highlights the exact correspondence between the two estimates.

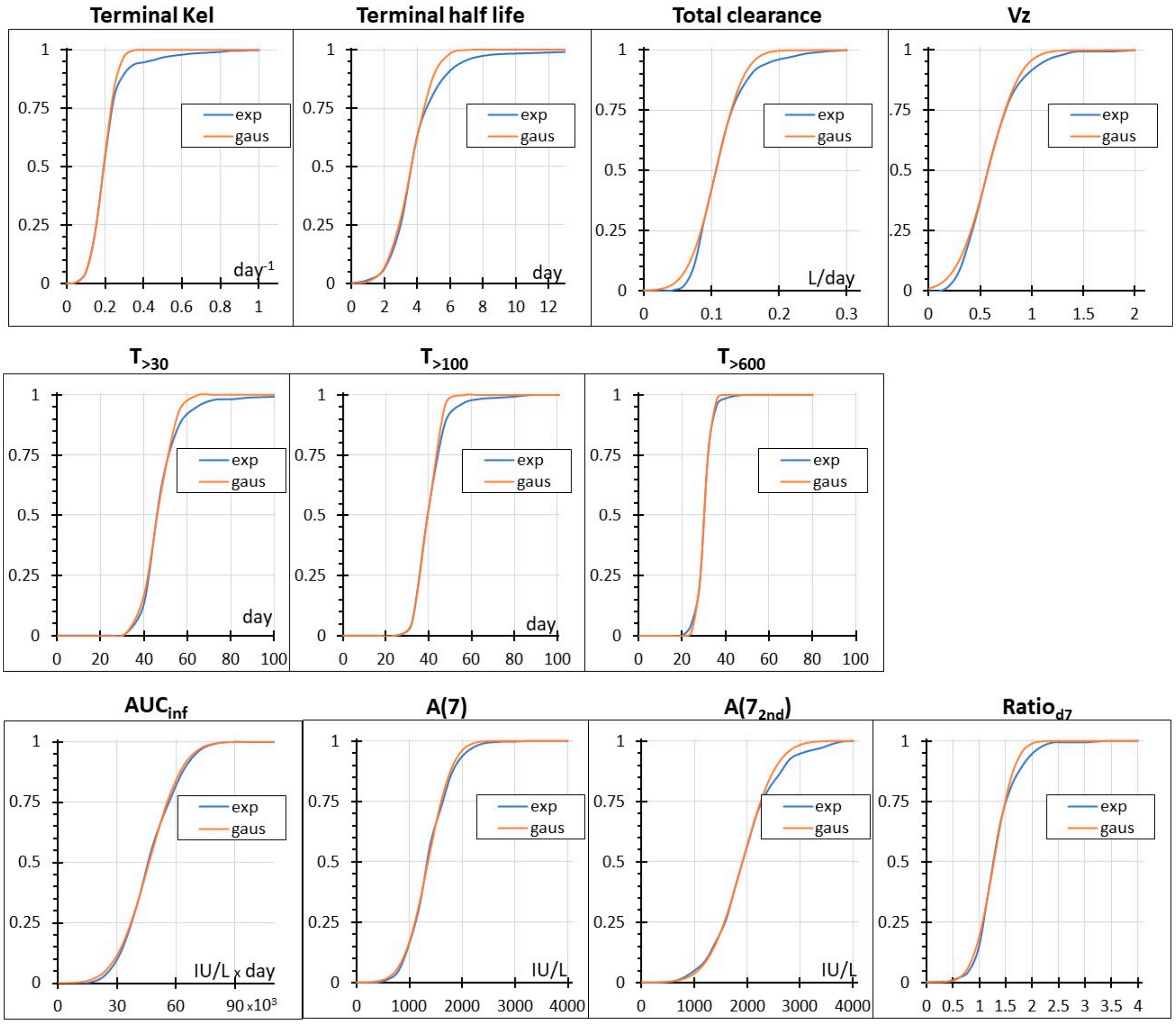

Figure S10. Cumulative distribution of experimental data (exp) and of the gaussian curve fitting the data between $p = 0.25$ (Q1) and $p = 0.75$ (Q3). Gaussians overlaps the experimental distribution in the fitting range, while a deviation from normality with observed above $p = 0.75$ (right tail) in *Kel*, T1/2, $T_{>30}$, $T_{>100}$, $T_{>600}$, Cl, Vz, A(72nd), Ratiod7, and below p=0.25 (left tail) in CL and Vz.

Table S1. Average values and variability range of exposure times above specific activity thresholds.

	Unit	N	Mean	CV	Median	IQR	5%-95% range
$T_{>30}$	day	426	47.9	21.6	45.9	9.0	35.2 - 65.5
$T_{>50}$	day	426	44.9	20.0	43.3	7.9	33.9 - 60.2
$T_{>100}$	day	426	41.0	17.6	39.6	6.2	32 - 53
$T_{>200}$	day	426	36.9	15.0	36.0	4.7	29.7 - 46.6
$T_{>400}$	day	426	32.7	12.7	32.2	3.8	27 - 39.8
$T_{>600}$	day	416	30.1	11.5	30.0	3.4	24.6 - 35.5
$T_{>800}$	day	398	27.8	11.5	28.0	4.3	21.9 - 32.5
$T_{>1000}$	day	350	25.8	13.0	26.3	4.6	19.8 - 30.5
$T_{>1500}$	day	144	22.7	13.7	22.7	4.2	16.8 - 27.7
